# Optimization of crystallization of biological macromolecules using dialysis combined with temperature control

**DOI:** 10.1107/S1600576720003209

**Published:** 2020-05-05

**Authors:** Niels Junius, Elham Vahdatahar, Esko Oksanen, Jean-Luc Ferrer, Monika Budayova-Spano

**Affiliations:** a Univ. Grenoble Alpes, CEA, CNRS, IBS, 38000 Grenoble, France

**Keywords:** crystal growth optimization, crystal size control, temperature–concentration phase diagrams, dialysis, temperature control, biomacromolecular crystallography

## Abstract

A rational way to find the appropriate conditions to grow crystal samples for bio-crystallography is to determine the crystallization phase diagram, which allows precise control of the parameters affecting the crystal growth process. First, the nucleation is induced at supersaturated conditions close to the solubility boundary between the nucleation and metastable regions. Then, crystal growth is further achieved in the metastable zone – which is the optimal location for slow and ordered crystal expansion – by modulation of specific physical parameters. Recently, a prototype of an integrated apparatus for the rational optimization of crystal growth by mapping and manipulating temperature–precipitant–concentration phase diagrams has been constructed. Here, it is demonstrated that a thorough knowledge of the phase diagram is vital in any crystallization experiment. The relevance of the selection of the starting position and the kinetic pathway undertaken in controlling most of the final properties of the synthesized crystals is shown. The rational crystallization optimization strategies developed and presented here allow tailoring of crystal size and diffraction quality, significantly reducing the time, effort and amount of expensive protein material required for structure determination.

## Abstract 2

This article describes rational strategies for the optimization of crystal growth using precise *in situ* control of the temperature and chemical composition of the crystallization solution through dialysis, to generate crystals of the specific sizes required for different downstream structure determination approaches.

## 1. Introduction   

**Figure 1:**
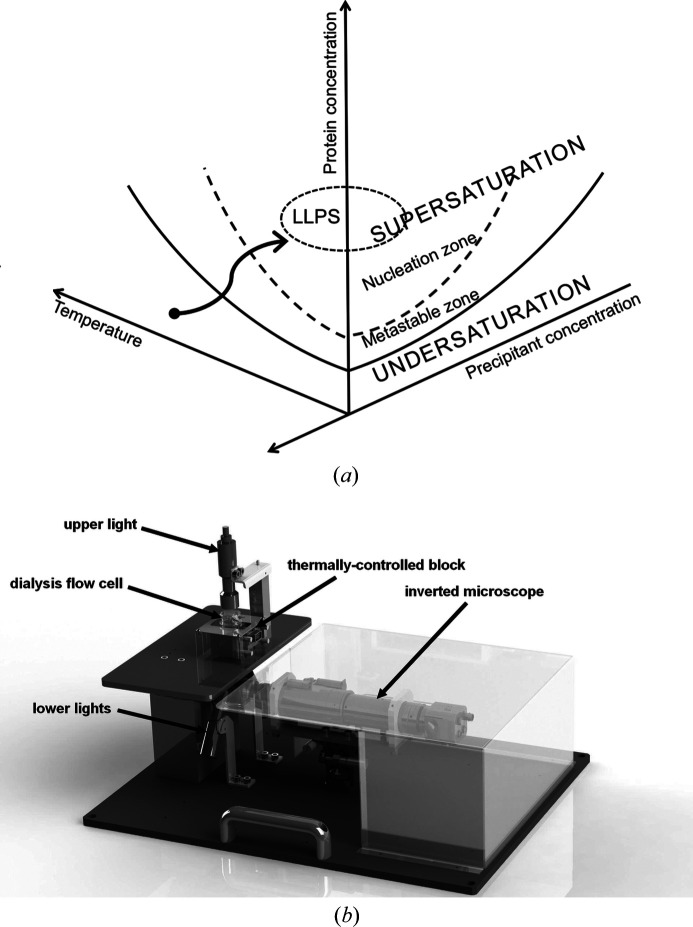
(*a*) Schematic view of a multidimensional phase diagram with two sections shown. The continuous curve is the solubility of the crystal phase as a function of temperature and the concentration of precipitant. The metastable zone lies between the solid and dashed curves, where the solution is supersaturated but nucleation of the crystal is either very slow or absent. Once the supersaturation is high enough, it drives nucleation and hence starts crystallization, represented by the nucleation zone, located next to the metastable zone. Finally, the process of liquid–liquid phase separation (LLPS), which occurs beyond the other zones, can both help and hinder crystallization. The arrow illustrates a specific kinetic pathway followed during crystallization. (*b*) Simplified view of the crystallization apparatus (OptiCrys) for temperature-controlled flow-cell dialysis with real-time visualization.

Knowledge of the phase diagram is of key importance when designing and controlling a crystallization process (Astier & Veesler, 2008 ▸; Vekilov, 2012 ▸; Zhang *et al.*, 2014 ▸). In the case of macromolecules such as proteins, the availability of accurate phase diagram data is limited owing to the diversity of their structures and the lack of suitable experimental setups to readily perform reliable measurements of protein solubility during crystallization using a small amount of protein sample (Garcia-Ruiz *et al.*, 1999 ▸; Olesberg *et al.*, 2000 ▸; Curtis *et al.*, 2001 ▸; Asherie, 2004 ▸; Dumetz *et al.*, 2007 ▸; Yin *et al.*, 2008 ▸; Y. Zhang *et al.*, 2012 ▸). The solubility of a protein depends strongly on the protein–protein interactions as well as on the protein–solvent interactions. Any slight modification of the solution composition can influence the solubility dramatically, or even alter the nature of these macromolecules. Independently of the complexity of protein behavior, the phase transformation is still governed by both the thermodynamics and the kinetics of the system. Therefore, it is still possible to describe all this information in phase diagrams. If crystallization conditions or nucleation points are identified, the information can be plotted in phase diagrams, as represented in a simplified form in Fig. 1 ▸(*a*). In this case the information provided relates to both thermodynamics and kinetics. The thermodynamic data are the solubility curves of the different crystalline and amorphous phases in the phase diagram. They depend on multiple parameters such as temperature, pH, solvent, impurities *etc.* In addition, kinetic trajectories in the phase diagram are relevant to control most of the final properties of the synthesized crystals. The path followed in the diagram controls the nucleation and growth of the crystals, and thus their number, size and morphology.

The success of diffraction experiments in protein crystallography is directly related to the quantity and quality of the recorded data. Considering the high performance of existing X-ray sources, it is now essentially the quality of the samples that limits the quality of the crystallographic data. New and emerging uses result in specific challenges for crystallization of proteins, in which precise control of crystal size is essential. New approaches to serial X-ray and electron crystallography, and to solving structures including time-resolved studies of short-lived intermediates, require small crystals, typically in the 0.2–10 µm size range. Serial crystallographic methods are being increasingly used at synchrotron sources (serial synchrotron crystallography) owing to advances in micro- and nano-focus beamlines, as well as at rapidly developing ultra-bright free-electron laser sources (serial femtosecond crystallography; Chapman, 2015 ▸), enabling structural studies of previously intractable proteins. Electron crystallography, traditionally applied to 2D membrane protein crystals, can now solve 3D structures from thin protein crystals and provide charge information not available from X-ray crystallography. This diffraction technique can potentially deliver atomic-resolution structural information with high throughput when suitable crystals (thinner than 200 nm for a typical 200 keV transmission electron microscope) are available (Nannenga & Gonen, 2016 ▸). At the other extreme are the requirements of the next-generation flagship neutron sources, such as the European Spallation Source (ESS, Lund). Because neutrons interact very weakly with matter, much larger, and ideally bulky, crystals are needed with volumes of >0.01 mm^3^ (*i.e.* 200 µm on a side) for neutron crystallography (Blakeley *et al.*, 2015 ▸). This is often the only way to visualize all of the protons in a protein structure, key information for the analysis of interactions required for drug design.

The need for detailed knowledge of the phase diagram is the basis of the devices (Budayova-Spano *et al.*, 2007 ▸; Budayova-Spano, 2010 ▸; Junius *et al.*, 2016 ▸) that we have developed with a focus on X-ray and neutron macromolecular crystallography. The first-generation instrument combines the use of temperature control and seeding and allows for growth of large crystals in a crystallization batch (Budayova-Spano *et al.*, 2007 ▸). A crystallization batch in the metastable zone is seeded with small protein crystals. The seeds are maintained inside this region of the phase diagram for as long as possible by adjusting the temperature each time the crystal-solution equilibrium is achieved. The temperature variations are repeated until crystals of suitable size for diffraction measurement are obtained. A dialysis button (Junius *et al.*, 2016 ▸), in addition to a crystallization batch, was later integrated into this instrument. This modification to the existing device (Budayova-Spano *et al.*, 2007 ▸) enables performing a temperature-controlled dialysis crystallization experiment.

The second-generation instrument, called the crystallization bench or OptiCrys [Fig. 1 ▸(*b*)], was built to allow the automation of the dialysis crystallization process (Junius *et al.* (2016 ▸). The instrument has recently been manufactured under license by the company NatX-ray (Saint Martin d’Hères, France). We added concentration control of the precipitant through the construction of a dialysis cell and a reservoir, allowing dialysis in continuous flow against a solution of controlled composition (Budayova-Spano, 2010 ▸; Junius *et al.*, 2016 ▸). Physico-chemical parameters such as temperature, concentration of crystallizing agents and pH can be controlled over time during the crystallization, so that the state of the substance studied moves along a well defined kinetic trajectory in the phase diagram. The dialysis membrane allows for adjusting the mass transfer through the membrane during the crystallization process. As a result, the gradients created can be controlled and affect the size and quality of generated crystals. Systematic phase diagrams in multidimensional space can be investigated using far less protein material than previously. With this serial approach, we mark a break with the current paradigm of parallel experiments. We demonstrate that established rational crystallization strategies can be beneficial to provide sufficient scattering volumes for neutron studies that require large-volume well ordered single crystals as well as to generate homogeneous populations of uniformly sized protein crystals required for use by other advanced serial diffraction techniques.

To validate the method beyond model systems like chicken egg-white lysozyme we have successfully tested the setup with a number of proteins of which large, single and well diffracting crystals were previously not available or were difficult to obtain. Altogether, in our validation tests with our apparatus, we have used six different proteins in order to demonstrate how to generate controlled-size crystals. Recombinant urate oxidase from *Aspergillus flavus* (Budayova-Spano, Bonneté *et al.*, 2006 ▸; Bonneté *et al.*, 2001 ▸) catalyses the oxidation of uric acid to allantoin. Neutron structure determination (Oksanen *et al.*, 2014 ▸) was based on the large crystals (Oksanen *et al.*, 2009 ▸) grown in the temperature-controlled batch setup (Budayova-Spano *et al.*, 2007 ▸). The fluorescent protein EosFP from *Lobophyllia hemprichii* (Wiedenmann *et al.*, 2004 ▸) undergoes a photoconversion upon irradiation with near-UV light and the crystals are therefore sensitive to light. Large and well diffracting crystals have been difficult to reproduce. Human carbonic anhydrase II catalyzes the reversible hydration of carbon dioxide. Large and well diffracting crystals for neutron diffraction (Budayova-Spano, Fisher *et al.*, 2006 ▸) were obtained with the temperature-controlled batch setup (Budayova-Spano *et al.*, 2007 ▸). Lactate dehydrogenase from *Thermus thermophilus* (Coquelle *et al.*, 2007 ▸) catalyses the last step in anaerobic glycolysis, conversion of pyruvate to lactate. Well diffracting but small crystals have been grown previously. YchB kinase from *Agrobacterium tumefaciens* (Borel *et al.*, in preparation) catalyses the first step of the isoprenoid biosynthetic pathway. It has been difficult to grow large single crystals of this enzyme.

## 2. Experimental procedures   

### 2.1. Description of the proteins and material used in crystallization   

Chicken egg-white lysozyme was purchased from Sigma–Aldrich as a lyophilized powder, dissolved in distilled water and filtered to obtain a solution with a final concentration of about 30 mg ml^−1^. Recombinant urate oxidase from *A. flavus* expressed in *Saccharomyces cerevisiae* was supplied by Sanofi–Aventis. A purine-type inhibitor (9-methyl uric acid) and the buffers, salts, PEGs and additives used in this study were purchased from Sigma–Aldrich. The protein complex with the inhibitor was prepared and crystallized according to the protocols described previously (Budayova-Spano, Bonneté *et al.*, 2006 ▸; Bonneté *et al.*, 2001 ▸). Crystallization solutions (Table 1 ▸) were prepared in both light and heavy water (Euriso-top, 99.92% D_2_O). The pD of the buffers was adjusted with NaOD (Euriso-top, 99% D) and DCl (Euriso-top, 99.8% D) according to the formula pD = pHmeas + 0.3314*n* + 0.0766*n* ^2^, where *n* = % D_2_O (Lumry *et al.*, 1951 ▸). The other proteins presented in our study were produced and purified following established protocols (Wiedenmann *et al.*, 2004 ▸; Budayova-Spano, Fisher *et al.*, 2006 ▸; Coquelle *et al.*, 2007 ▸; Borel *et al.*, in preparation). All protein concentrations were measured by UV absorbance at 280 nm (Table 1 ▸). All the solutions were filtered through 0.22 µm Millipore filters. In all the crystal growth experiments, the crystallization mixtures were obtained using dialysis techniques (Ducruix & Giégé, 1992 ▸). The cellulose membranes used in our experiments were the standard RC membrane Spectra/Por (http://spectrumlabs.com) with molecular weight cut-offs (MWCOs) of 6–8 kDa and 12–14 kDa. Before the start of the experiment, the crystallization mixtures were centrifuged and filtered to remove all solid particles (precipitates, dust or nuclei). Details of the physico-chemical properties and crystal growth conditions of the proteins studied here are summarized in Table 1 ▸.

### 2.2. Crystallization setups   

In the semi-automated first-generation version of the instrument, the protein solution is poured into a specially designed stainless steel dialysis chamber with a transparent polycarbonate optical bottom separated from the precipitant solution by a dialysis membrane of the appropriate molecular weight cut-off (Junius *et al.*, 2016 ▸). The dialysis membrane is placed over the top of the dialysis chamber containing the sample (a variety of sizes of 25–200 µl are available) and is held in place by a groove in the dialysis button with an elastic ring. A stainless steel well is then placed over the dialysis button and plays the role of a reservoir containing the precipitant (Junius *et al.*, 2016 ▸). This dialysis setup is temperature-controlled using Peltier elements, and the variation of the chemical composition of crystallization solution in the reservoir during the experiment is performed manually (Budayova-Spano *et al.*, 2007 ▸). In the new, second-generation version of the instrument, the dialysis button is replaced by a new fluidic assembly composed of the dialysis chamber, located on the bottom, with the reservoir chamber on the top, connected to a pumping system functioning as a continuous flow cell (Junius *et al.*, 2016 ▸). This setup enables the exchange of the chemical composition in an automated way during the crystallization experiment. The user may adjust the composition of the reservoir solution and hence access different parts of the phase diagram of the molecule to be crystallized. Any combination of precipitant concentration and temperature can be explored in a systematic manner by sampling a continuum of potential crystal-producing conditions without physically perturbing the mother liquor, while the total volume of protein solution remains constant during the entire experiment. The rate of diffusion through the dialysis membrane can be controlled by using membranes with a particular molecular weight cut-off. The flow-cell dialysis setup is inserted into a brass support temperature-controlled using Peltier elements and is incorporated into the microscope table for viewing of the crystallization chamber from below by an inverted microscope [Fig. 1 ▸(*b*)]. Illumination is provided by light-emitting diodes [Fig. 1 ▸(*b*)]. The operating temperature range of 233–353 K ± 0.1 K is reached using a proportional–integral–derivative electronic temperature controller. The Peltier elements are cooled on one face with a chiller, resulting in improved temperature control, and a circuit of a dry air prevents condensation. The control software is written with *LabVIEW* (http://www.ni.com/labview/) and includes a graphical user interface for visualization and measurement of crystals, image acquisition, processing and storage as well as control of each parameter (temperature control, illumination, pumping the solutions and measuring the concentration of the different constituents of the crystallization solution).

### 2.3. Crystal growth optimization workflow   

**Figure 2:**
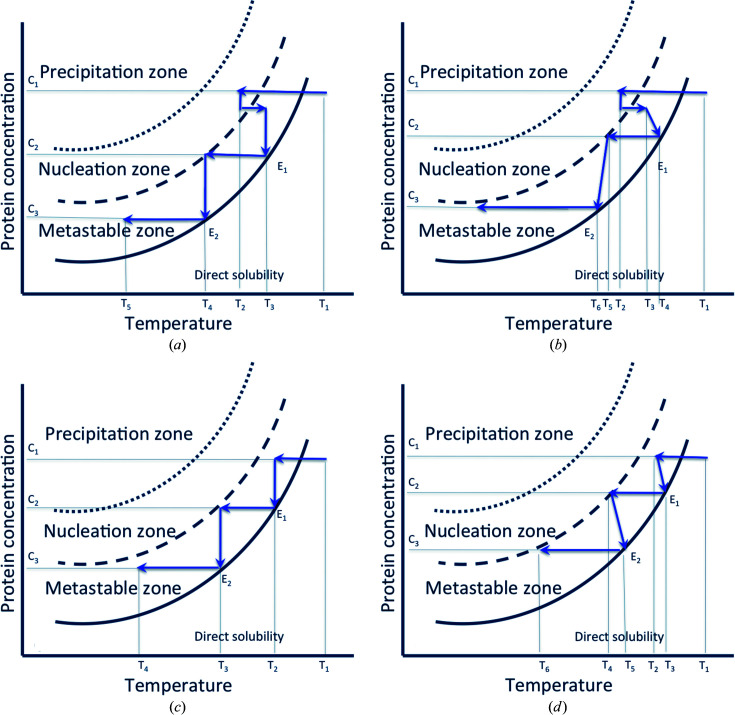
Principle of the method of crystallization with temperature changes at a constant concentration of crystallizing agent. Kinetic pathways schematizing the standard kinetic trajectory A1, resulting in the induction of nucleation in the zone of the spontaneous nucleation and respective crystal growth in the metastable zone due to the controlled temperature variations (*a*), and other alternative workflows A2, showing additional temperature variations covering the stages of arrival at equilibrium in workflow A1 (*b*), and induction of the crystal growth of seeded crystals in the metastable zone as a result of the controlled temperature variations (*c*), supplemented by additional temperature variations covering the stages of arrival at equilibrium (*d*).

**Figure 3:**
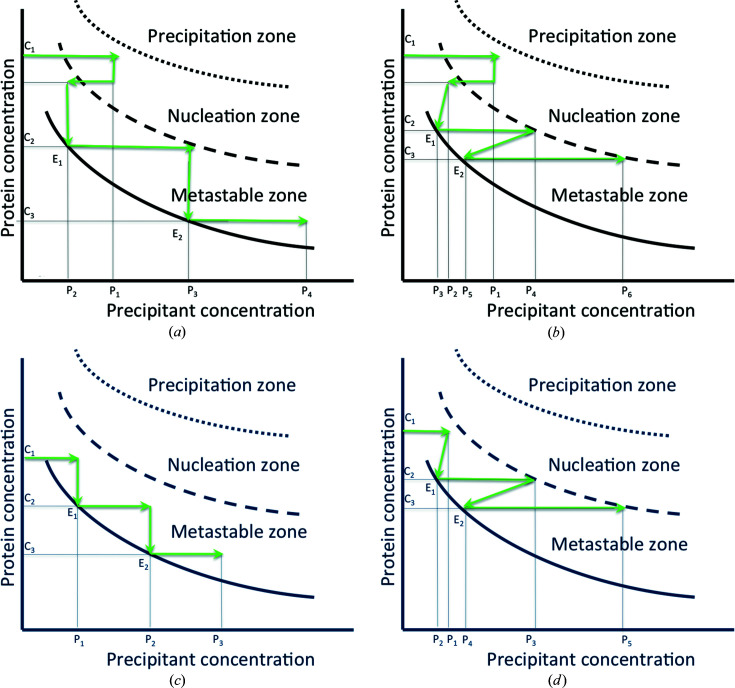
Principle of the method of crystallization at constant temperature with variations in concentration of crystallization agent. Kinetic pathways schematizing the standard kinetic trajectory B1, resulting in the induction of nucleation in the zone of spontaneous nucleation and respective crystal growth in the metastable zone due to the controlled variations of the precipitant concentration (*a*), and other alternative workflows B2, showing additional precipitant concentration variations covering the stages of arrival at equilibrium in workflow B1 (*b*), and induction of the crystal growth of seeded crystals in the metastable zone as a result of the controlled precipitant concentration variations (*c*), supplemented by additional precipitant concentration variations covering the stages of arrival at equilibrium (*d*).

In this section we describe the principle of the method of crystallization with temperature changes at a constant concentration of crystallizing agent (Fig. 2 ▸) and at constant temperature with variations in concentration of crystallization agent (Fig. 3 ▸). In each optimization scheme, the case of the standard kinetic trajectory resulting in the induction of nucleation in the zone of spontaneous nucleation and respective crystal growth in the metastable zone is shown [Figs. 2 ▸(*a*) and 3 ▸(*a*)]. Since other alternative workflows are possible, the standard kinetic pathway is then supplemented by further variations in temperature (or precipitant concentration) covering the equilibrium arrival steps [Figs. 2 ▸(*b*) and 3 ▸(*b*)]. Figs. 2 ▸(*c*) and 3 ▸(*c*), respectively, illustrate the case of the induction of crystal growth of crystals seeded in the metastable zone by controlled temperature variations and concentration of precipitant. This last case is finally completed by additional variations in temperature or concentration of precipitant, covering the stages of arrival at equilibrium [Figs. 2 ▸(*d*) and 3 ▸(*d*)].

*Standard trajectory A1*. The process of optimizing crystal growth for a solute – such as a protein – as a function of temperature (T) at a constant precipitant concentration (P) in the case of direct solubility (solubility increases with the temperature), starting from known crystallization conditions, has the following steps (Fig. 2 ▸):

(1) The crystallization chamber is filled with protein solution at a first temperature T1 at a solute concentration C1. The reservoir is filled with a precipitant solution of concentration P1, which slowly diffuses into the crystallization chamber through the dialysis membrane [Fig. 2 ▸(*a*)].

(2) The temperature is decreased to T2 in order to drive the kinetic trajectory in the phase diagram to the spontaneous nucleation zone [Fig. 2 ▸(*a*)] or up to the vicinity of the upper limit of the metastable zone, to induce nucleation.

(3) The temperature is increased to T3, which stops nucleation, and the trajectory leads to the metastable zone of the phase diagram.

(4) The crystals are left to grow at T3 until a first equilibrium point E1 is reached [Fig. 2 ▸(*a*)], where the size of the crystals remains constant and the concentration of the solute decreases to the concentration C2.

(5) The temperature is then decreased to T4, which is within the metastable zone of the phase diagram so no new nucleation occurs.

(6) The crystals are left to grow at T4 until a second equilibrium point E2 is reached [Fig. 2 ▸(*a*)] where the crystals no longer grow and the solute concentration reaches C3.

(7) Steps 4 to 6 are repeated until crystals of the desired size are obtained.

(8) The crystals are harvested.

When a homogeneous population of small crystals (controlled by the supersaturation level chosen for nucleation) is desired, steps 3–6 are not cycled. During a cooling crystallization the crystal size is governed by the relative rates of nucleation and growth. These in turn are driven thermodynamically by the level of supersaturation. It is worth considering that the rate at which supersaturation is increasing with time (the supersaturation rate) is as important as the supersaturation value. It is also dependent on the diffusion of protein around the crystals and therefore on the mass transport in the crystallization cell (García-Ruiz *et al.*, 2016 ▸). At high supersaturation levels, nucleation tends to dominate, giving rise to a preponderance of smaller crystals. At low supersaturation levels, growth tends to dominate, resulting in fewer but larger crystals.

Other alternative workflows are possible:

*Alternative trajectory A2*. All the steps previously described as the stages of temperature variation (steps 1, 2, 3 and 5) are repeated as described above, and the stages of arrival at equilibrium (steps 4 and 6) will be drawn here also with a temperature variation [shown in Fig. 2 ▸(*b*)]. In this alternative, the dialysis chamber houses a protein solution pre-equilibrated with the crystallization agent at low supersaturations corresponding to the metastable zone of the phase diagram. A small seed of crystal will be placed before the flow-cell dialysis setup is permanently closed. Then the growth of the seed crystal can also be illustrated according to the workflows described above, by inducing the temperature variations in steps 4, 5 and 6 [Fig. 2 ▸(*c*)]. It will also be possible to induce additional temperature variations covering the stages of arrival at equilibrium [shown in Fig. 2 ▸(*d*)].

*Trajectory B1*. A similar process can be used to optimize crystal growth at a constant temperature T as a function of a precipitant concentration P (Fig. 3 ▸). The steps here are the following:

(1) A protein solution in the crystallization chamber of the flow-cell dialysis setup, at a concentration C1, is driven to the spontaneous nucleation zone or up to the vicinity of the upper limit of the metastable zone of the phase diagram by a precipitant solution of concentration P1 in the reservoir, thus inducing nucleation [Fig. 3 ▸(*a*)].

(2) The concentration of the precipitant is lowered to P2, to stop nucleation and direct the system into the metastable zone of the phase diagram.

(3) The crystals are allowed to grow at P2 until a first equilibrium point E1 is reached [Fig. 3 ▸(*a*)], where the crystals no longer grow and the solute concentration is C2.

(4) The precipitant concentration is increased to P3, still within the metastable zone so no further nucleation occurs.

(5) The crystals are allowed to grow at P3 until a second equilibrium point E2 is reached [Fig. 3 ▸(*a*)], where the crystals no longer grow and the solute concentration is C3.

(6) Steps 3 to 5 are repeated until crystals of the desired size are obtained.

(7) The crystals are harvested.

As in the previous case, when a homogeneous population of small crystals (controlled by the level of supersaturation chosen for nucleation) is desired, steps 2–5 are not cycled. During crystallization by increasing the ionic strength, the crystal size is governed by the relative rates of nucleation and growth and these are in turn controlled thermodynamically by the level of supersaturation. However, the supersaturation rate is as important as the supersaturation value and is also dependent on the diffusion of protein molecules around the crystals and therefore on the mass transport in the crystallization cell (García-Ruiz *et al.*, 2016 ▸). At high levels of supersaturation, nucleation will dominate, giving rise to a preponderance of smaller crystals. At low levels of supersaturation, growth will dominate, giving rise to larger and fewer crystals.

As in the previous case, other basic alternative workflows are possible:

*Alternative trajectory B2.* All the steps previously described as the stages of precipitant concentration variation (steps 1, 2 and 4) are repeated as described above and the stages of arrival at equilibrium (steps 3 and 5) will be drawn here also with a supplemental variation [represented in Fig. 3 ▸(*b*)]. In this alternative, the dialysis chamber houses a protein solution pre-equilibrated with the crystallization agent at low supersaturations corresponding to the metastable zone of the phase diagram. A small seed of crystal will be placed before the flow-cell dialysis setup is permanently closed. Then the growth of the seed crystal can also be illustrated according to the workflows described above, by inducing the concentration variations of a precipitant in steps 1, 2 and 4 [Fig. 3 ▸(*c*)]. It is also possible to induce additional concentration variations of a precipitant covering the stages of arrival at equilibrium [shown in Fig. 3 ▸(*d*)].

Variations of these basic workflows can be easily envisaged, for example changing both temperature and precipitant concentration either sequentially or simultaneously. In this case the kinetic trajectory in the phase diagram is best described in three dimensions. To control the growth of ordered crystals while avoiding additional nucleation we search for conditions approaching the limit of the metastable zone of the three-dimensional phase diagram. The variation in the size of the crystals is measured, for example by taking photographs of the crystals approximately every 20–30 min when the growth of the crystal begins, and then every 2–3 h towards the end. The pictures are then processed by means of an image analysis software package. The approach described above might be spread over a time period of up to two months, in particular for large crystal volumes for neutron protein crystallography. For X-ray protein crystallography, a time period from 1 to 2 weeks is typically sufficient. The selection of the temperatures during these steps depends on the nature of the molecules to be crystallized and on their thermal stability. The optimal ranges of the temperature for the different protein systems studied here are summarized in Table 1 ▸.

## 3. Results and discussion   

Various empirical approaches, based on screening and optimization, have been proposed to generate crystals using vapor diffusion, batch crystallization, dialysis, seeding, free-interface (or counter) diffusion and temperature-induced crystallization. Some of these methods make use of high-throughput automated instrumentation and miniaturization of crystallization experiments and have huge impacts on protein crystallization in terms of saving time and conserving precious sample [*e.g.* Microcapillary Protein Crystallization System (Gerdts *et al.*, 2008 ▸); Fluidigm Corporation TOPAZ system (Segelke, 2005 ▸); *in meso* crystallization robot (Cherezov *et al.*, 2004 ▸); automated microseed matrix screening (D’Arcy *et al.*, 2007 ▸); microlytic Crystal Former (Stojanoff *et al.*, 2011 ▸)]. Several approaches have also been developed to automate crystal detection from the imaged drops (Echalier *et al.*, 2004 ▸; Forsythe *et al.*, 2006 ▸; Groves *et al.*, 2007 ▸) and others to simplify the identification of crystallization hits (Judge *et al.*, 2005 ▸; Dierks *et al.*, 2008 ▸; Kissick *et al.*, 2011 ▸).

### 3.1. Crystallization optimization experiments   

The first crystallization experiments presented here were performed using the temperature-controlled dialysis buttons that were incorporated into the crystal growth apparatus of the first-generation instrument (Budayova-Spano *et al.*, 2007 ▸).

**Figure 4:**
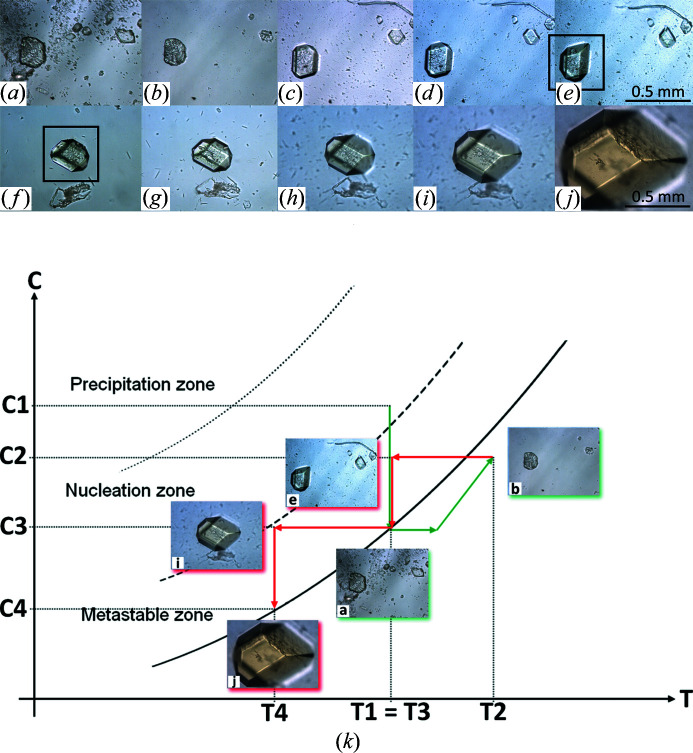
Dialysis experiment with variation of the temperature in the case of growth of large crystals of the urate oxidase complex with 9-methyl uric acid. (*a*) Situation at the end of the third day. Once the concentration of the crystallization agent inside the dialysis button at T1 = 293 K has reached that of the reservoir solution, the formation of many small crystals of the Uox–9MUA complex is observed next to a few seeded crystals in the dialysis button. The temperature is increased to T2 = 298 K. (*b*) Situation after 10 min from dissolution of small crystals in excess at T2 = 298 K. Growth of a few crystals observed at T3 = 293 K during the first day (*c*) and 2 days after dissolution (*d*). (*e*) Growth of a few crystals and of one selected seeded crystal observed at T3 = 293 K 5 days after dissolution. Growth of the selected seeded crystal observed (*f*) 5 days, (*g*) 7 days and (*h*) 9 days at 293 K and (*i*) 12 days and (*j*) 17 days at T4 = 291 K after dissolution. (*k*) Schematic phase diagram (protein concentration versus temperature) incorporating some selected images (to be tracked in alphabetical order) to illustrate the optimization workflow in growing large Uox–9MUA complex crystals. In accordance with alternative workflow A2 (Section 2.3), C1 represents the initial protein concentration used in the crystallization experiment (8 mg ml^−1^). C2 is weaker than the initial protein concentration, as it results from crystal growth in the metastable zone. C3 and C4 are then protein concentrations corresponding to the relative equilibrium points reached.

In agreement with the alternative workflow A2 (Section 2.3), large crystals of the recombinant urate oxidas (Uox) from *A. flavus* in complex with 9-methyl uric acid (9MUA) were grown from a few crystals (Fig. 4 ▸) seeded in the pre-equilibrated mother liquor [protein solution containing 1–2%(*w*/*v*) of PEG 8K] before final equilibration to 5%(*w*/*v*) was reached in the dialysis chamber (Table 1 ▸). After 3 days at 293 K, many small crystals grew next to the few larger crystals of the previously seeded Uox–9MUA complex [Fig. 4 ▸(*a*)]. To reduce the number of crystals in the dialysis button, the temperature was then raised to 298 K for 10 min to dissolve the excess of small crystals [Fig. 4 ▸(*b*)]. It was subsequently reduced to 293 K and to 291 K to promote the growth of 2–3 selected crystals of interest, including the selected crystal shown in the remainder of the sequence in Fig. 4 ▸. Finally, large crystals of the Uox–9MUA complex were obtained by following the kinetic pathway illustrated in the schematic equilibrium phase diagram represented in Fig. 4 ▸(*k*).

**Figure 5:**
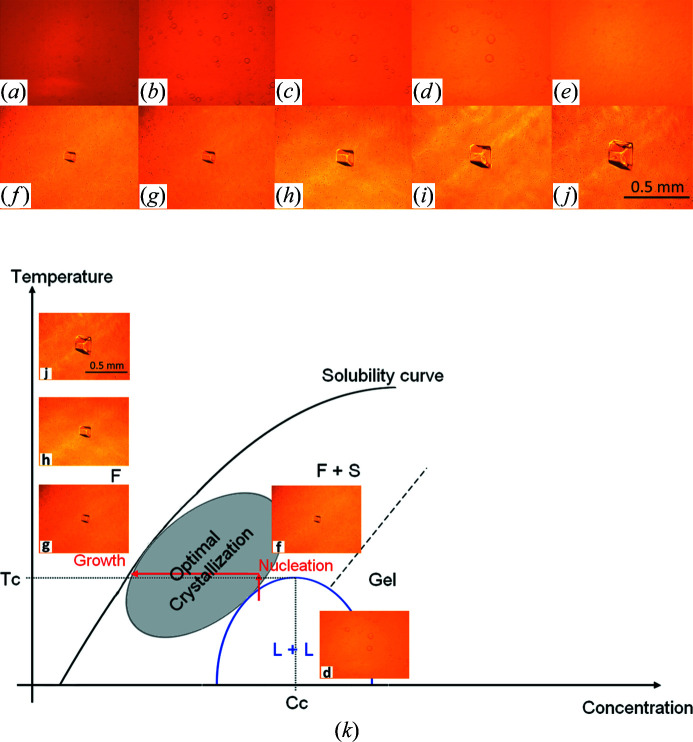
The dialysis experiment with variation of the temperature in the case of crystal growth of EosFP from the process of LLPS. (*a*) Approximately 2 h after injection of the protein into the dialysis button at 293 K, the formation of the dense liquid droplets of the protein-rich phase emerging from the LLPS in the dialysis is observed. The temperature is increased to 295.5 K. Dissolution of the dense liquid droplets at 295.5 K (*b*) 30 s later, (*c*) 1 min later, (*d*) 90 s later and (*e*) 2 min later. (*f*) Approximately 6 h later at 295.5 K, the appearance of the first crystal formed as a result of the LLPS process is observed. Growth of the formed crystal at 295.5 K (*g*) 12 h later, (*h*) 36 h later, (*i*) 3 days later and (*j*) and 6 days later. (*k*) Schematic, temperature versus protein concentration, conceptual construct of the phase diagram incorporating some selected images (to be tracked in alphabetical order), showing the optimization workflow in growing EosFP crystals from the LLPS process. Various zones are shown: areas of the fluid (protein solution or F), coexistence of the protein-rich and protein-poor liquid phases (LLPS or L + L), coexistence of the crystal (S) and the protein solution (F + S), possible gelation (Gel), and optimum condition for protein crystallization (Optimal Crystallization). T_C_–C_C_ represents the critical point beyond which LLPS is not longer possible.

With fluorescent protein EosFP we demonstrated that temperature variation can be successfully used to induce nucleation resulting from the process of (metastable) liquid–liquid phase separation (LLPS) (Fig. 5 ▸). After 2 h at 293 K, 2 *M* ammonium sulfate (Table 1 ▸) had completely diffused into the dialysis button and the demixing of two liquids (protein-rich and protein-poor liquid phases) was observed [Fig. 5 ▸(*a*)]. The temperature was then raised to 295.5 K. Photographs (*b*), (*c*), (*d*) and (*e*) in Fig. 5 ▸ show the transformation of the dense liquid droplets of the protein-rich phase during the first 2 min that followed the increase in temperature in the crystallization chamber. The dense liquid droplets dissolved and after 6 h the first crystal appeared [Fig. 5 ▸(*f*)]. The sequence of photographs [Figs. 5 ▸(*g*), 5 ▸(*h*), 5 ▸(*i*) and 5 ▸(*j*)] as well as the corresponding schematic conceptual construct of the phase diagram [Fig. 5 ▸(*k*)] show the crystal growth at 295.5 K resulting from the LLPS process.

**Figure 6:**
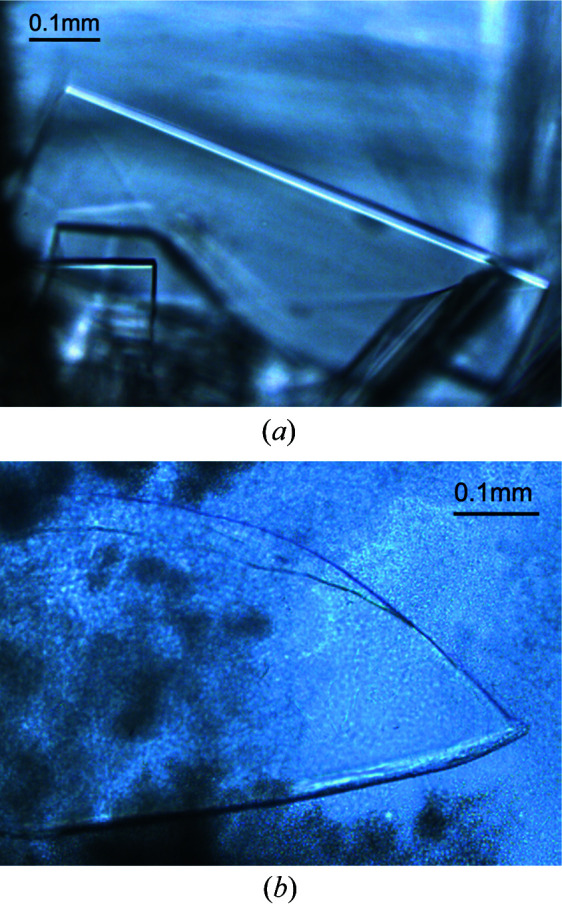
Crystals of human carbonic anhydrase II (*a*) and YchB kinase from *A. tumefaciens* (*b*) obtained in about 1 week in the temperature-controlled dialysis button with volumes of 25 and 70 µl, respectively.

Two other proteins that crystallized successfully using the temperature-controlled dialysis buttons were human carbonic anhydrase II (hCA II) and YchB kinase from *A. tumefaciens* (Table 1 ▸). The crystals were obtained in about 1 week (Fig. 6 ▸). In the case of hCA II – by following the standard workflow B1 described in Section 2.3 – the concentration of ammonium sulfate was varied by dialysis from 0.8 to 1.2 *M* every 2 days with increments of 0.2 *M* at a constant temperature of 278 K [Fig. 6 ▸(*a*)]. In the case of YchB, the situation was the opposite since the standard workflow A1 described in Section 2.3 was used. In this case, the concentration of the precipitant PEG 8000 was kept constant, at 20%(*w*/*v*), and the temperature was changed from 295 to 293 K at the end of the fourth day, 2 days after the equilibrium had been reached in the dialysis button [Fig. 6 ▸(*b*)].

**Figure 7:**
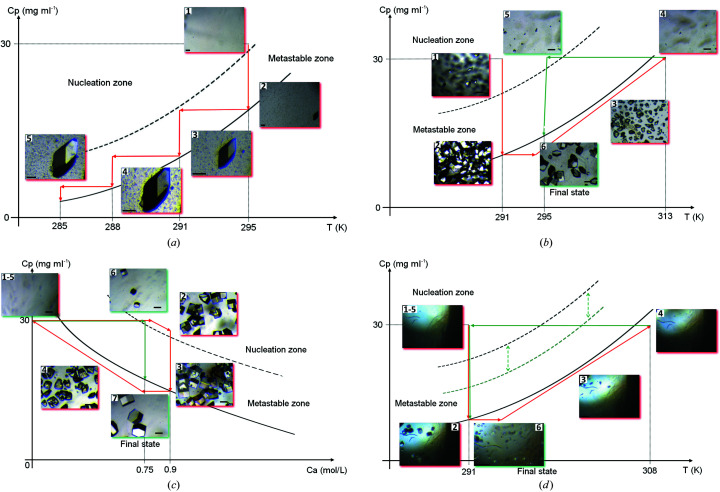
Schematic phase diagrams (protein concentration versus temperature or precipitant concentration) incorporating some selected images (to be tracked in ascending order) and illustrating the crystallization optimization workflow in growing chicken egg-white lysozyme crystals: (*a*) Generating the large single lysozyme crystal obtained at constant chemical composition using the control of the temperature variations. (*b*) Generating uniformly sized lysozyme crystals at constant chemical compositions using the control of the temperature variations. (*c*) Generating uniformly sized lysozyme crystals at constant temperature using the control of the concentration of crystallizing agent. (*d*) Generating a large number of small uniformly sized lysozyme crystals obtained at constant chemical composition using the control of the temperature variations. The shrinkage of the metastable zone by increasing the rate generating the supersaturation is shown schematically. (Scale on photographs represents 100 µm.)

Other examples of crystallization trials presented here were carried out with the prototype temperature-controlled dialysis flow-cell setup (second-generation instrument) to demonstrate the control of nucleation and crystal growth. The first example (Fig. 7 ▸) is a proof-of-principle experiment with chicken egg-white lysozyme (Table 1 ▸), demonstrated in our previous work (Junius *et al.*, 2016 ▸) and completed here with one extra experiment. As described previously, protein solution was placed in the crystallization chamber of the temperature-controlled dialysis flow cell. Fig. 7 ▸(*a*) reproduces the controlled process demonstrated for large-crystal growth of a single lysozyme crystal (Junius *et al.*, 2016 ▸). The pictures incorporated in the qualitative crystallization phase diagram show the crystal habit and volume of a nucleated crystal observed before equilibration at 295 K (after 3 days), at 291 K (after 1 day), at 288 K (after 4 days) and at 285 K (after 2 days). Ten days after the start of the experiment, the overall crystal growth process was complete, leading to a large single crystal of the volume typically needed in neutron protein crystallography when a protein is perdeuterated (around 0.1 mm^3^). Next, Figs. 7 ▸(*b*) and 7 ▸(*c*) demonstrate the reversibility of the dialysis experiments for nucleation, crystal growth, dissolution and re-growth, where fewer but larger lysozyme crystals resulted from temperature and precipitant concentration variations, respectively (Junius *et al.*, 2016 ▸). This is schematically illustrated from our previous experiment with the help of qualitative crystallization phase diagrams that incorporate pictures showing the crystal habit and size of nucleated crystals in first and second nucleation events. In these two cases, the lysozyme crystals obtained during the first nucleation event are more numerous and smaller [photographs surrounded by red in Figs. 7 ▸(*b*) and 7 ▸(*c*)] than those obtained during the second nucleation event [photographs surrounded by green in Figs. 7 ▸(*b*) and 7 ▸(*c*)] induced by the variation of the temperature [Fig. 7 ▸(*b*)] or the concentration of precipitant [Fig. 7 ▸(*c*)], the latter being visibly fewer and larger. Finally, the last experiment with lysozyme [Fig. 7 ▸(*d*)], carried out under the conditions detailed in Table 1 ▸ and launched at 291 K, illustrates the opposite case: nucleation, crystal growth, dissolution, and re-nucleation and growth of a large number of very small crystals of lysozyme. After 120 min (the estimated time for diffusion of NaCl to the crystallization chamber is 90 min) the first crystals appeared, and a few days later, near to the equilibrium at 291 K, crystals with a size of around 50 µm had grown in the dialysis chamber. Then the temperature was increased to 308 K in order to dissolve all crystals [photographs surrounded by red in Fig. 7 ▸(*d*)]. After more than 24 h at 308 K, when dissolution was almost complete, we again lowered the temperature to the initial value at 291 K. This time, after a few hours, a large number of crystals with very small volume, with size less than 10 µm, appeared at the bottom of the crystallization chamber [photograph surrounded by green in Fig. 7 ▸(*d*)].

**Figure 8:**
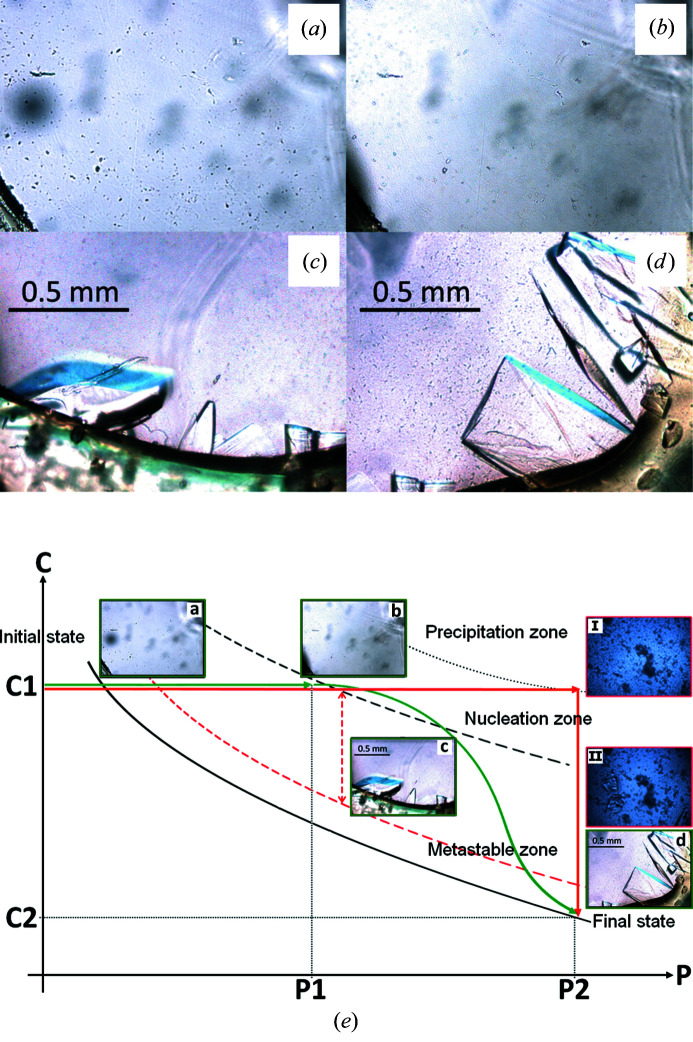
The dialysis crystallization experiment with lactate dehydrogenase from *T. thermophiles* carried out at 293 K. (*a*) The beginning of the crystallization experiment (time 0). (*b*) Three days later, when the PEG 6000 concentration in the crystallization chamber is P1 = 2.5%(*w*/*v*). (*c*), (*d*) Two days after the PEG 6000 concentration was increased to P2 = 5%(*w*/*v*), crystals of the enzyme are observed and grow to large volume. (I) Situation observed in the comparative dialysis experiment carried out directly at P2 = 5% once the concentration difference between the compartments has been reached and (II) a few days later at equilibrium. (*e*) Schematic phase diagram (protein concentration versus precipitant concentration) incorporating selected images (to be tracked in ascending or alphabetical order) and illustrating the crystallization optimization workflow in growing large lactate dehydrogenase crystals. The enlargement of the metastable zone by reducing the rate generating the supersaturation is shown schematically. In accordance with a variant (not shown in Fig. 4 ▸) of the standard workflow B1 (Section 2.3), C1 represents the initial protein concentration used in the crystallization experiment (2.8 mg ml^−1^) at P1 and C2 is the protein concentration corresponding to the relative equilibrium point reached at P2.

The last example presented here is lactate dehydrogenase from the hyperthermophilic bacterium *T. thermophilus*. In this case, the dialysis experiment was carried out at constant temperature, *T* = 293 K, but with a variation in composition of the crystallization agent. Protein solution at a concentration of *ca* 2.8 mg ml^−1^ was placed in the crystallization chamber of the temperature-controlled dialysis flow-cell setup. The composition of the reservoir solution was varied from 2.5 to 5%(*w*/*v*) of PEG 6000 in 100 m*M* MES buffer at pH 6 with an increment of 2.5%(*w*/*v*) of PEG (Fig. 8 ▸). Initially [Fig. 8 ▸(*a*), time 0] the PEG concentration inside the crystallization chamber was 0%. Three days later, the concentration of PEG inside the dialysis button reached that of the reservoir at 2.5%. No crystals were observed at this point [Fig. 8 ▸(*b*)]. The PEG concentration in the reservoir was then increased to 5%(*w*/*v*) and 2 days later large crystals appeared [Fig. 8 ▸(*c*) and 8 ▸(*d*)].

In order to rationalize these experiments, we performed some comparative dialysis experiments with human carbonic anhydrase II and lactate dehydrogenase from *T. thermophilus*. The same batches of proteins were used (30 mg ml^−1^ in the case of hCA II and 2.8 mg ml^−1^ in the case of lactate dehydrogenase). The main difference between these experiments and those presented previously is the fact that here we do not impose an extra precipitant concentration or a temperature gradient. The precipitant concentration in the reservoir was kept constant and corresponds to the final supersaturation where the nucleation took place in previous experiments [1.2 *M* ammonium sulfate, 100 m*M* Tris–HCl pH 8.6 in the case of hCA II and 5%(*w*/*v*) PEG 6000, 100 m*M* MES pH 6 in the case of lactate dehydrogenase], and the temperature was maintained at 293 K. After a few days, a crystalline precipitate was observed at the bottom of the dialysis chamber in the case of lactate dehydrogenase, as shown in the pictures I and II, surrounded in red, in Fig. 8 ▸(*e*), illustrating a schematic kinetic pathway imposed during the crystallization process in the corresponding phase diagram. In the case of hCA II a huge number of small crystal clusters grew from such conditions. Finally, another interesting comparison concerns crystallization experiments conducted under the same crystallization conditions with YchB kinase from *A. tumefaciens* using the temperature-controlled dialysis button with temperature variation and traditional vapor diffusion techniques at constant temperature. We were unable to obtain single crystals of YchB kinase using the traditional vapor diffusion techniques. Only large twinned crystals or crystal clusters could be grown by vapor diffusion, whereas the temperature-controlled dialysis method yielded large single crystals. The bound adenosine triphosphate molecules were also clearly visible in the electron density map, unlike the crystals grown with vapor diffusion (Borel *et al.*, in preparation).

### 3.2. Temperature effect   

Temperature is recognized as a non-invasive control parameter for protein crystallization. Before temperature-induced crystallization can be routinely used as a method of preparation of protein crystals, qualitative data on the temperature-dependent solubility of the protein should be obtained. However, it is only available for a limited number of proteins, because of the labor-intensive techniques required for the experimental determination of solubility curves. Knowledge of the phase diagram and the specific control of the crystallization parameters such as the temperature and the concentration of crystallization agents and/or additives as described here allow the number of crystals and their macroscopic defects to be reduced, as well as selection of the nucleation and/or growth of the desired phase. Even when the precise position of the solubility curve of the phase diagram is not experimentally known, the ability to control the crystallization parameters in a reversible manner together with real-time observation of the crystals allows the phase diagram to be explored in a qualitative way.

Temperature or precipitant gradients can be used to precisely and reversibly control the relative supersaturation levels of protein solutions. Temperature controls the balance between enthalpy and entropy effects on free energy, which are comparable in magnitude. Depending on whether crystallization is enthalpy driven or entropy driven, proteins become either more soluble at higher temperatures (direct solubility) or less soluble at higher temperatures (reverse solubility) (Budayova-Spano *et al.*, 2007 ▸; Oksanen *et al.*, 2009 ▸). The temperature influence is due to variation of the acid/base constants of the protein side chains (Chernov & Komatsu, 1995 ▸). In addition, the effective pK_a_ values of the ionizable groups are related to the ionic strength of the medium. As a result, the solubility increases with temperature when the ionic strength is low (*i.e.* the solution contains low dielectric constant components) and *vice versa*. The temperature–solubility function is not a property of the protein itself, but is linked to the protein-solution system. Therefore, the choice of the buffer is crucial and should not alter the ionic strength of the system as far as possible. In addition, depending on the buffer substance, its pH may vary with temperature. As an example, the Tris buffer, being probably the most frequently used buffer in biological experiments, is not always the best choice since it has a significantly high degree of temperature sensitivity. The effects are therefore very different when Tris is used at 277 K, at room temperature or at 310 K. This means that the pH value has to be set for the temperature around which it is used.

Here we demonstrated the kinetic ripening method, previously successfully employed in growth of large protein crystals by the batch crystallization technique (Budayova-Spano *et al.*, 2007 ▸) and reported also by other authors (Astier & Veesler, 2008 ▸) (Fig. 4 ▸), in an experiment in which the crystal size distribution is wide due to nucleation [Fig. 4 ▸(*a*)]. In contrast to an isothermal Ostwald ripening (a phenomenon occurring at constant temperature), here the temperature fluctuations imposed in the neighborhood of the equilibrium temperature induce dissolution of the smallest crystals and growth of the largest with the same phase. The pictures of crystals in Fig. 4 ▸ and the corresponding theoretical phase diagram illustrated in Fig. 4 ▸(*k*) present the complete kinetic ripening process for the Uox–9MUA complex. In the first stage, temperature is increased by a few degrees. Small and large crystals dissolve [Fig. 4 ▸(*b*)] in the undersaturated zone in the vicinity of the solubility curve [pictures surrounded by green in Fig. 4 ▸(*k*)], but as small crystals have less matter to be transferred they dissolve faster and the process is stopped before complete dissolution of the larger crystals by a temperature decrease (second stage). Finally, large faceted crystals grow [Figs. 4 ▸(*c*)–4(*j*)] inside the metastable zone [pictures surrounded by red in Fig. 4 ▸(*k*)].

Protein precipitates are often considered as a dead-end product that cannot evolve towards monocrystals, but in fact many macromolecules have been observed to crystallize from precipitates. It is now clearly established that these precipitates consist of aggregates or gels produced by metastable LLPS (Broide *et al.*, 1996 ▸; Grouazel *et al.*, 2002 ▸; Asherie, 2004 ▸; Vivarès & Bonneté, 2004 ▸; Dumetz *et al.*, 2008 ▸). The conceptual construct of the phase diagram (temperature versus protein concentration) illustrates the liquid–liquid coexistence curve (blue line) in the case of fluorescent protein EosFP [Fig. 5 ▸(*k*)]. The coexistence curve shows the boundary between the region where the protein solution remains homogeneous and the region where demixing occurs and dense droplets with protein-rich phase form, leading to LLPS. According to Ostwald’s rule of stages, LLPS occurs prior to crystal nucleation: for kinetic and thermodynamic reasons, liquid nucleation, which proceeds by density fluctuation alone, is faster and easier than crystal nucleation, which requires both density and structure fluctuation (Vekilov, 2010 ▸). In practice, an increase in temperature or a decrease in protein concentration leads to supersaturated conditions in which droplets of the dense phase dissolve [Figs. 5 ▸(*a*)–5(*e*)]. This zone in the T–C phase diagram, below the solubility curve and above the LLPS curve, represents the location where the right crystallization conditions can be found. Fine-tuning the temperature therefore leads to better control of nucleation and growth. This example shows that even qualitative knowledge of the phase diagram and the respective positions of the phase boundaries for LLPS and crystal nucleation allow identification of optimal conditions for protein crystallization. Our experimental design allowed us to induce crystal nucleation from the liquid–liquid separation of metastable phases by changing the temperature, which is consistent with the literature (Broide *et al.*, 1996 ▸). Another way to induce crystallization from the LLPS process would be to change the composition of the crystallization solution at constant temperature and at constant protein concentration (F. Zhang *et al.*, 2012 ▸).

The described approaches can be also beneficial in the case of twinned crystals or crystal clusters as mentioned for YchB kinase from *A. tumefaciens*, where the temperature-controlled dialysis crystallization enabled us to obtain large single crystals unlike the traditional method of vapor diffusion. The presence of multiple phases such as polymorphs, solvates, microcrystalline solids of other phases and amorphous liquids may complicate growth of the desired crystal phase. Here again, the controlled fluctuations of temperature and/or of concentrations of crystallization agents and/or additives may be used to drive the transition phenomenon of the phases (disappearance of one phase to the benefit of another phase). Such solution-mediated phase transitions can be used to grow large crystals of the stable phase at the expense of the metastable phase as demonstrated previously (Budayova *et al.*, 1999 ▸; Oksanen *et al.*, 2010 ▸). The same applies for the macroscopic defects of the crystals such as satellite crystals bonded onto single crystals which can be completely dissolved to the benefit of the growth of large single crystals (Budayova-Spano *et al.*, 2007 ▸; Astier & Veesler, 2008 ▸). The crystal habit and crystal quality are also improved because different growth conditions (*e.g.* temperature and supersaturation) induce different crystal growth rates for each face (Budayova *et al.*, 1999 ▸; Budayova-Spano *et al.*, 2007 ▸; Junius *et al.*, 2016 ▸).

### 3.3. Crystallization kinetics   

The metastable zone width is an essential parameter for the growth of large-size crystals from solution, since it is a direct measure of stability of the solution in its supersaturated region. The larger the zone width, the higher the stability (Buckley, 1951 ▸; Zaitseva *et al.*, 1995 ▸). It appears that our apparatus and crystallization procedure enabled us to modify crystallization kinetics by reducing the equilibration rates and thus to provide better control of crystal nucleation and crystal growth compared with the current methods widely used in protein crystallization. The comparative crystallization experiments with and without variation of the chemical composition of the reservoir in the cases of lactate dehydrogenase and of hCA II demonstrate clearly that precipitant concentration gradients affect the nucleation kinetics. Successively increasing the crystallizing agent concentration in the dialysis crystallization process, in contrast to the traditional dialysis experiment in which the gradient is not imposed, will result in a decrease of the rate of generating the supersaturation and therefore the reduction of nucleation rate and the growth of fewer and larger crystals. The interpretation of this phenomenon with the phase diagram is that the kinetic extent of the metastable zone is enlarged and therefore the spontaneous nucleation rate is decreased at higher supersaturations by the precipitant concentration gradient. This is illustrated by the theoretical phase diagram representing the case of lactate dehydrogenase from *T. thermophilus* [Fig. 8 ▸(*k*)]. As mentioned in our previous work, this kind of crystal-growth process is very beneficial for neutron studies that require large single crystals to provide sufficient scattering volumes (0.1–1.0 mm^3^, depending on the unit-cell volume and deuteration approach).

On the other hand, the crystal growth rate is a function of supersaturation, that is, the higher the supersaturation, the higher the growth rate. As already mentioned, nucleation or formation of tiny crystals is also driven by the supersaturation. Consequently, monitoring the level of supersaturation over time helps to control the growth and nucleation rates achieved, thereby controlling the size of the crystals produced. This has been demonstrated here with a model protein, chicken egg-white lysozyme. We were able to drive the crystallization process to generate the desired number and size of crystals in all crystallization experiments. In particular, here we emphasize the case where a large number of very small crystals have been generated [Fig. 7 ▸(*d*)]. In agreement with the theory, the temperature variations that we induced during this experiment led to the shrinkage of the metastable zone (unlike that observed in the case of hCA II or lactate dehydrogenase) of the corresponding phase diagram [Fig. 7 ▸(*d*)]. Therefore, the relatively rapid decrease in temperature to restart nucleation [picture surrounded by green in Fig. 7 ▸(*d*)] leads to an increase in the rate generating the supersaturation. Nucleation is fast, many crystals form nearly simultaneously and the majority of crystals grow to approximately identical size. This kind of crystal growth process may be beneficial for free-electron laser and synchrotron serial crystallography, which require large numbers of uniformly sized small crystals (<50 µm).

Note that all the examples shown in this work have a direct solubility as a function of temperature and make use of the salting-out regime of the protein–precipitant phase diagram. However, our methodology is well suited to also deal with inverse solubility and make use of the salting-in regime, which is somewhat underutilized in growing large crystals.

## 4. Conclusion   

Given the time and effort involved in material preparation and structure determination, it is justified to devote more time to optimizing the crystalline material, since the quality or size of the crystals often limits what can be learned. Here we describe rational protocols based on multidimensional crystallization phase diagrams that we established for *in situ* generation of crystals of specific sizes and morphology optimized for different downstream structure determination approaches using our recently developed device (OptiCrys). This approach is illustrated by the crystallization of several soluble proteins. OptiCrys consists of crystal growth apparatus that, in addition to precise temperature monitoring, also allows the control and change of the crystallization solution components (*e.g.* precipitant concentration, buffer, additive, ligands) in an automated manner through dialysis. The crystallization process can be monitored and controlled in real time with a video microscope and a PC via supervision software. The system allows the survey of multiple crystallization conditions in a systematic way with the same biological sample. The sample is not consumed in the experiment, and if the sample is not damaged (*e.g.* denatured), the conditions can be changed reversibly. This enables one to optimize the kinetic path through the phase diagram, which controls the nucleation and growth of the crystals, and thus their number, size and morphology. Tailoring of crystal number, size, phase and diffraction quality reduces the time, protein material and efforts required for structure determination. The described strategy differs from the current paradigm in performing serial instead of parallel experiments. The dialysis membrane acts as a precision dosing device to control the composition of the crystallization solution, the level of supersaturation and its generation rate by tuning the mass transfer across the membrane. Mass transport in crystallization experiments is the process that forms supersaturation gradients and therefore affects both crystal size and diffraction quality. As demonstrated from diffraction quality of lysozyme and YchB kinase crystals grown as described here and already tested (Junius *et al.*, 2016 ▸), the transfer of solutes and solvent through semipermeable membranes seems to provide the slowly varying conditions required for early crystal nucleation and relatively undisturbed structure formation in crystal growth. In the future, we aim to carry out these studies on other protein targets in order to assess crystal mosaicity as well as homogeneity of the crystal structure by appropriate methods, such as X-ray diffraction topography.

## Figures and Tables

**Table 1:** Summary of some physico-chemical properties of the proteins used and their crystal growth conditions

Protein system	MW (kDa)	pI	Crystallization condition	Protein concentration (mg ml^−1^)	Membrane MWCO (kDa)	Dialysis volume (µl)	Temperature range (K)
Urate oxidase from *A. flavus*	33.8	Basic 7.5	5%(*w*/*v*) PEG 8000, 100 m*M* NaCl, 50 m*M* Tris–HCl pD 8.5	8	6–8	100	278–298
Chicken egg-white lysozyme	14.3	Basic 11.35	0.75 *M* sodium chloride, 100 m*M* sodium acetate pH 4	30	6–8	45	278–313
Fluorescent protein EosFP from *L. hemprichii*	25.8	Acidic 6.9	2 *M* ammonium sulfate, bicine 100 m*M* pD 8	18	6–8	25	278–295
Lactate dehydrogenase from *T. thermophilus*	32.8	Acidic 5.8	5%(*w*/*v*) PEG 6000, 100 m*M* MES pH 6	2.8	6–8	15	278–293
Human carbonic anhydrase II	30	Acidic 6.8	1.2 *M* sodium citrate, 100 m*M* Tris–HCl pH 8.6	30	6–8	25	278–313
YchB kinase from *A. tumefaciens*	31.8	Acidic 6.1	20%(*w*/*v*) PEG 8000, MgCl_2_ 5 m*M*, ATP 5 m*M*, sodium citrate pH 6	10	12–14	70	278–295
